# Highly Efficient and Stable CdZnSeS/ZnSeS Quantum Dots for Application in White Light-Emitting Diode

**DOI:** 10.3389/fchem.2022.845206

**Published:** 2022-03-08

**Authors:** Xi Chen, Jingzhou Li, Yichi Zhong, Xin Li, Mingzhong Pan, Hongxing Qi, Hongxing Dong, Long Zhang

**Affiliations:** ^1^ Shanghai Institute of Technical Physics, Chinese Academy of Sciences, Shanghai, China; ^2^ University of Chinese Academy of Sciences, Beijing, China; ^3^ Hangzhou Institute for Advanced Study, University of Chinese Academy of Science, Hangzhou, China; ^4^ Shanghai Institute of Optics and Fine Mechanic, Chinese Academy of Sciences, Shanghai, China

**Keywords:** CdZnSeS/ZnSeS, quantum dots, light-emitting diode, stability, white light-emitting diodes

## Abstract

Semiconductor quantum dots (QDs) are a promising luminescent phosphor for next-generation lightings and displays. In particular, QD-based white light-emitting diodes (WLEDs) are considered to be the candidate light sources with the most potential for application in displays. In this work, we synthesized quaternary/ternary core/shell alloyed CdZnSeS/ZnSeS QDs with high bright emission intensity. The QDs show good thermal stability by performing high temperature-dependent experiments that range from 295 to 433 K. Finally, the WLED based on the CdZnSeS/ZnSeS QDs exhibits a luminous efficiency (LE) of 28.14 lm/W, an external quantum efficiency (EQE) of 14.86%, and a warm bright sunlight close to the spectrum of daylight (Commission Internationale de l'éclairage (CIE) coordinates 0.305, 0.371). Moreover, the photoluminescence (PL) intensity, LE, EQE, and correlated color temperature (CCT) of as-prepared QD WLED remained relatively stable with only slight changes in the luminescence stability experiment.

## Introduction

Semiconductor quantum dots (QDs) have shown emerging significant promise as solid-state lightings and displays (Li et al., 2020; Zvaigzne et al., 2020), sensors (Koeppel et al., 2007; Liang et al., 2021), biomedicine (Zvaigzne et al., 2016; Pashazadeh-Panahi and Hasanzadeh, 2019), biological labeling (Bai et al., 2020), solar cell (Xu T. et al., 2021; Ostadebrahim and Dehghani, 2021), and laser physics (Nautiyal et al., 2021; Tsuji et al., 2021) owing to their superior optoelectronic properties such as high photoluminescence (PL) quantum yield (QY) (PLQY), narrow emission bandwidth, size-controlled tunable emission wavelength, and high photochemical stability and durability (Zhang et al., 2015; Li Q. et al., 2016; Harris et al., 2016; Leach and Macdonald, 2016; Giansante and Infante, 2017; Owen and Brus, 2017; Ghosh and Manna, 2018). In particular, QDs are considered to be the candidate materials with the most potential for application in the next generation of lightings and displays (Moon and Chae, 2020; Fang et al., 2021; Kıbrıslı et al., 2021; Li et al., 2021; Yuan et al., 2021). As the core materials of light-emitting diode (LED) devices based on the QDs, high luminescence efficiency and stable luminescence properties are one of the crucial factors for large-scale commercialization. However, when QDs are irradiated by strong light for a long time or the temperature of the QD device is high due to the resistance of the electric circuit, the brightness of the QDs often becomes unstable or even dim. Intense efforts have been carried out to solve the stability of the QD device by changing the surface composition, structure, ligand, solvent, etc. (Xie et al., 2018; Li et al., 2019; Zhou et al., 2019; Xu Y. et al., 2021).

Among various QDs, II–VI compounds have attached extensive interest, such as CdSe, CdZn, and CdZnSe. To passivate non-radiative surface states and extend the emission spectral coverage, coating with another semiconductor shell with a relatively wide bandgap is one of the alternative means (Regulacio and Han, 2010; Jia and Tian, 2014). Especially, multicomponent alloy QD materials have attracted more and more attention owing to their excellent stability and optical properties. First, the ternary alloyed QDs, for example, CdZnS (Liu et al., 2013) and CdZnSe (Sheng et al., 2014), can be prepared. But the emission peaks of the ternary CdZnS or CdZnSe QDs are limited from 400 to 620 nm, which restricts them from achieving high color rendering index (CRI) for a white LED (WLED). Several groups (Adegoke et al., 2016; Nasrin et al., 2020) reported water-soluble quaternary/ternary core/shell alloyed CdZnSeS/ZnSeS QDs via tuning and controlling the sulfur molar fraction (ternary shell layer). The QDs exhibited a remarkable PLQY of 36%–98% and good stability. However, systematic studies on the thermal stability of the quaternary alloy QDs have been relatively rare, and the high-quality WLEDs based on the multicomponent alloy QDs still need to be further explored.

Here, the quaternary/ternary alloyed CdZnSeS/ZnSeS QDs with two different emission colors were synthesized by the thermal injection method. In temperature-dependent experiments, the QDs show good thermal stability, with the redshift of only 36.33 meV from 295 to 433 K. The WLED based on the prepared CdZnSeS/ZnSeS QDs exhibits excellent optical performances and high luminous stability. We demonstrate the superior optical properties of quaternary/ternary alloyed CdZnSeS/ZnSeS QDs and show the prospect as WLED luminescent material.

## Experimental

Similar to the previously reported synthesis procedure, the quaternary/ternary alloyed CdZnSeS/ZnSeS QDs were fabricated by the hot-injection method (Adegoke et al., 2016). First, a mixture of 1.3 g of CdO, 0.6 g of hexadecylamine (HAD), 50 ml of octadecene (ODE), and 30 ml of oleic acid (OA) was loaded into a 3-neck flask, which was stirred and heated to 280°C under N_2_. As the temperature of the solution approached 260°C, trioctylphosphine (TOP) (2.23 ml) and Se/TOP (12 ml) precursors were injected into the Cd-HAD-OA solution to initiate the nucleation and growth of the binary CdSe seeds. Furthermore, the precursors of Se/TOP (12 ml), ZnO/OA (20 ml), and S/trioctylphosphine oxide (TOPO) (50 ml) were added into the complex solution above to initiate the nucleation and growth of the quaternary CdZnSeS QDs in succession. The solutions were taken at different times to record UV–Vis absorption and PL emission spectra of the QDs. After the alloyed core QDs were achieved, a solution of ZnO, S/TOP, and Se/TOP precursors was added swiftly for the overcoating of the ternary ZnSeS shell layer. The obtained QDs in crude solution were separated by centrifuging. After centrifugation, the quaternary/ternary alloyed CdZnSeS/ZnSeS QDs were redispersed in toluene.

The LED chip with the emission peak at 385 nm was used for the fabrication of WLED. The as-prepared green-emitting and red-emitting CdZnSeS/ZnSeS QDs (15 mg/ml) were mixed homogeneously with polymethyl methacrylate (PMMA) (0.1 mg/ml, dissolve into toluene). To avoid self-absorption of green emission light, the PMMA solution containing the red QDs was first coated on the LED chip, and then the mixture with the green QDs was deposited. At the same time, each step was cured at 60°C for 10 min.

Micro-morphologies of the CdZnSeS/ZnSeS QDs were observed via transmission electron microscopy (TEM) (Talos F200X G2, Thermo Fisher Scientific, Waltham, MA, USA). The absorption spectra were recorded at room temperature ranging from 300 to 800 nm using a UV–Vis spectrophotometer (V-770, JASCO, Oklahoma City, OK, USA). The PL spectral and PL delay curves were measured on a steady-state and time-resolved PL spectrometer (FLS1000+FS5). The temperature-dependent PL experiments were performed using a fluorescence spectrometer (FluoroMax-4) with a high-temperature fluorescence controller (TAP-02) at the temperature range from 295 to 433 K. The PLQY, luminous efficiency (LE), external quantum efficiency (EQE), emission spectrum, correlated color temperature (CCT), and Commission Internationale de l'éclairage (CIE) of QD WLED were studied systematically.

## Result and Discussion

**FIGURE 1:**
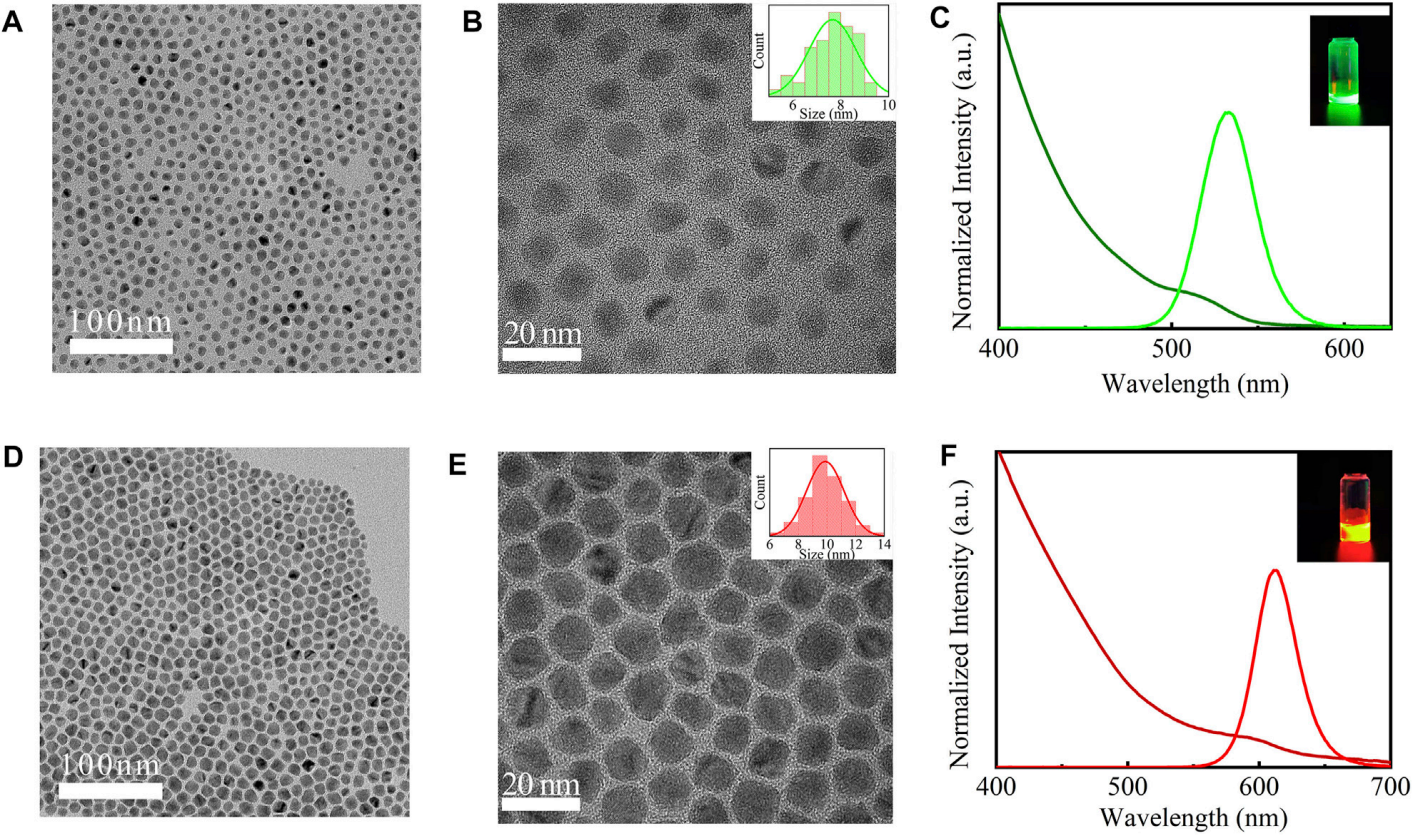
TEM images of the CdZnSeS/ZnSeS QDs. **(A,B)** CdZnSeS/ZnSeS QDs at green emission. **(D,E)** CdZnSeS/ZnSeS QDs at red emission. **(C** and **F)** PL and UV–Vis absorption spectra for the QDs at room temperature. TEM, transmission electron microscopy; QDs, quantum dots; PL, photoluminescence.

**FIGURE 2:**
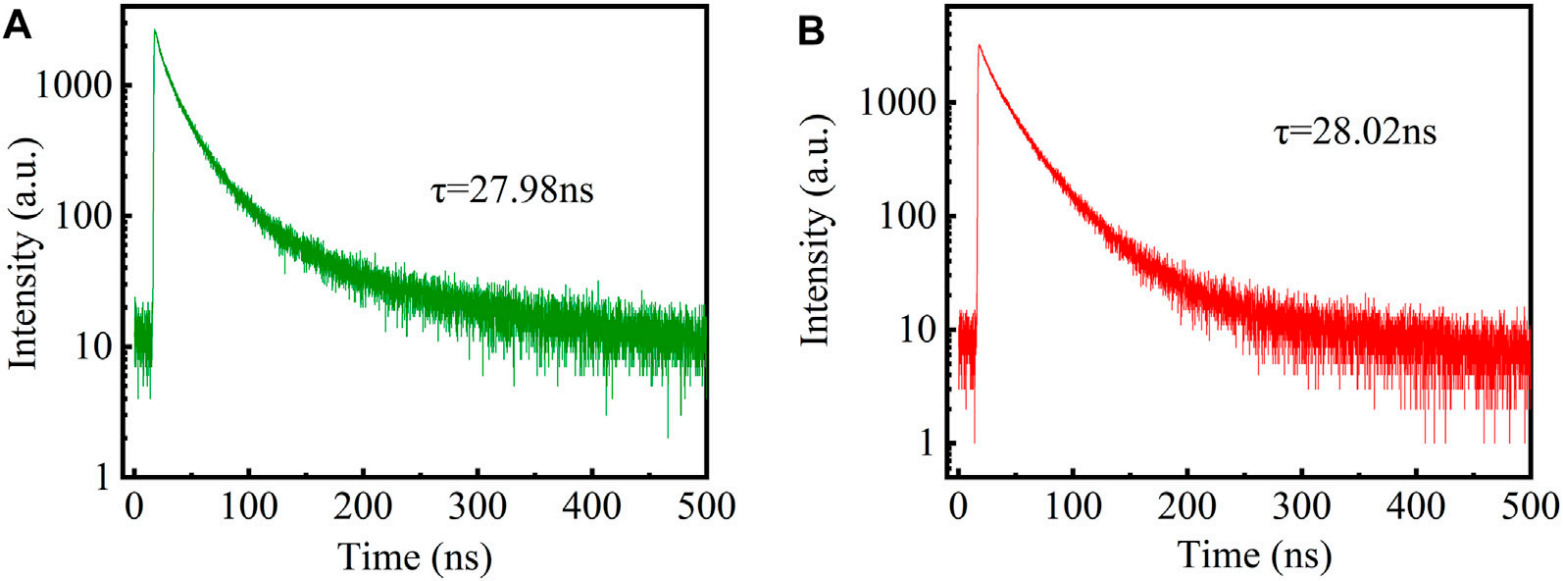
PL decay curves of the CdZnSeS/ZnSeS QDs at room temperature. **(A)** Green QDs. **(B)** Red QDs. PL, photoluminescence; QDs, quantum dots.

As shown in Figures 1A–E, the quaternary/ternary alloyed CdZnSeS/ZnSeS QDs exhibited good monodispersion and a nearly spherical shape. Nano Measure software was used to estimate the particle size distribution of the sample. The size of the green CdZnSeS/ZnSeS QDs is concentrated at about 7 nm (Figures 1A,B). The size of the red CdZnSeS/ZnSeS QDs is about 9 nm (Figures 1D,E). Figures 1C,F show the absorption spectra and PL emission spectra at room temperature of CdZnSeS/ZnSeS QDs. Here, the band-edge absorption peak of the green QDs was 513 nm (2.41 eV). The PL spectrum showed a full width at half maximum (FWHM) of about 36 nm (160 meV) and was centered at 532 nm (2.33 eV), which corresponded to a non-resonant Stokes shift of 19 nm (80 meV). The band-edge absorption peak of the red QDs was 595 nm (2.08 eV). The PL spectrum showed an FWHM of about 37 nm (123 meV) and was centered at 613 nm (2.02 eV), which corresponded to a non-resonant Stokes shift of 18 nm (60 meV). As shown in the inset of Figures 1C,F, the emitting light of the CdZnSeS/ZnSeS QDs exhibits bright light. The PLQY of the QDs is measured to be 63% (green QDs) and 51% (red QDs). Figure 2 shows representative PL decay curves of the green QDs and red QDs. The results are fitting based on biexponential decay functions, for which the calculated average decay time of the green QDs is 27.98 ns, and the red QDs is 28.02 ns.

**FIGURE 3:**
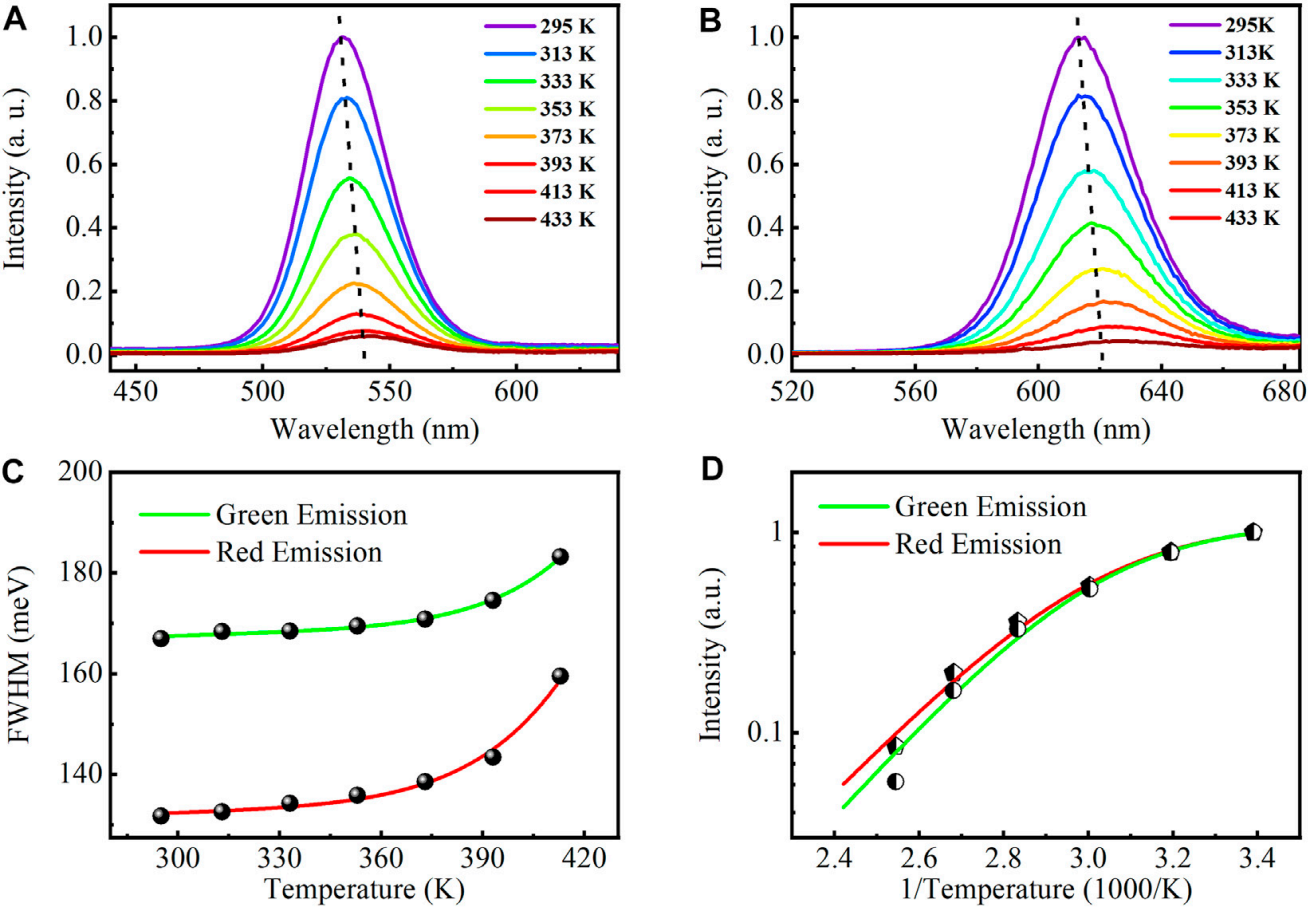
Photoluminescence spectra of the CdZnSeS/ZnSeS QDs at various temperatures (T = 295 K–433 K). **(A)** CdZnSeS/ZnSeS QDs at green emission. **(B)** CdZnSeS/ZnSeS QDs at red emission. Temperature-dependent change of **(C)** FWHM and **(D)** intensity. QDs, quantum dots; FWHM, full width at half maximum.

To investigate the thermal stability of the CdZnSeS/ZnSeS QDs, we performed variable temperature experiments using a high-temperature fluorescence controller at the range from 295 to 433 K. The PL spectrum was measured by fluorescence spectrometer with an excitation light source at 361 nm. Figures 3A,B show the temperature-dependent PL spectrum of the CdZnSeS/ZnSeS QDs. We can find that the emission peaks have redshift with the increase of temperature. The PL intensities of the two samples decreased significantly with the increase in the temperature. At the same time, the broadening of spectra was observed. As shown in Figure 3A, the PL intensity of the green CdZnSeS/ZnSeS QDs at 433 K is 91.1% lower than that at 295 K. The redshift is 32.26 meV from 295 to 433 K. As shown in Figure 3B, the PL intensity of the red CdZnSeS/ZnSeS QDs at 433 K is 92.4% lower than that at 295 K. However, the redshift of the red QDs is basically the same as that of green QDs, from 295 to 433 K. According to the PL emission–excitation intensity relationship, the small redshift of the samples with the increase of temperature implies that the quaternary/ternary alloyed CdZnSeS/ZnSeS QDs have a stronger excitonic character with fewer defect states (Chen et al., 2019; Hien et al., 2020), which demonstrated that the synthesized samples have high quality.

The exciton–phonon coupling of the quaternary/ternary alloyed CdZnSeS/ZnSeS QDs is studied by analyzing the FWHM energy of emission spectra as a function temperature. Figure 3C shows the PL FWHM of the CdZnSeS/ZnSeS QDs at various temperatures. The FWHM of the QD PL spectrum is consistently broadened with the temperature increase from 295 to 433 K, which is close to the inhomogeneity of QD size and the scattering between excitons and optical/acoustic phonons. The experimental data of PL linewidth broadening are well fitted with the following equation (Wright et al., 2016):

$$\begin{array}{c} \Gamma \left(T\right)=\Gamma_{inh}+\theta T+\Gamma_{LO}{\left(e^{E_{LO}/k_{B}T}-1\right)}^{-1} \end{array}$$ (1)

where $\Gamma_{\mathrm{inh}}$ corresponds to the temperature-independent inhomogeneous broadening, which arises from scattering due to impurities and imperfections; θ is the acoustic-exciton–phonon interaction coefficient; $\;\Gamma_{\mathrm{LO}}$ is the longitudinal optical (LO)-exciton–phonon coupling coefficient; $E_{\mathrm{LO}}$ is the phonon energy; and $\;k_{B}$ is the Boltzmann constant. The temperature dependence of the FWHM energy for CdZnSeS/ZnSeS QDs can be fitted well, as shown in Figure 3C. The parameters $\Gamma_{\mathrm{inh}}$ are calculated to 125 meV (green QDs) and 161 meV (red QDs). The small value of $\Gamma_{\mathrm{inh}}$ indicates the uniform size distribution of the particle, which is consistent with the TEM result. The LO-phonon energy ( $E_{\mathrm{LO}}$ ) for two of the QDs were calculated to be 575 meV (green) and 643 meV (red), respectively, indicating strong exciton–phonon interactions (He et al., 2019, 3; Hamada et al., 2020). This is significantly higher than the previous binary QD materials (for example, CdSe/ZnS QDs ∼24 meV) (Chang et al., 2021).

Furthermore, to analyze PL thermal quenching, the dependence of PL spectral emission intensity with temperature is studied. The Arrhenius equation is used to fit these results (Wu et al., 1996):

$$\begin{array}{c} I\left(T\right)=\frac{I\left(0\right)}{1+Ae^{-E_{b}/k_{B}T}}\;\; \end{array}$$ (2)

where I(T) is the integrated PL intensity at temperature T, I(0) represents the intensity at low temperature, $E_{b}$ is the effective binding energy, and **A** is a constant. $E_{b}$ is an intrinsic physical parameter for semiconductors. The parameter $E_{b}$ was obtained to be 0.364 meV (green) and 0.333 meV (red), which is suggested as originating in the transformation of longitudinal acoustics phonon. This value is significantly lower than the previous binary QD materials (for example, CdSe/ZnS QDs ∼50 meV) (Li J. et al., 2016).

**FIGURE 4:**
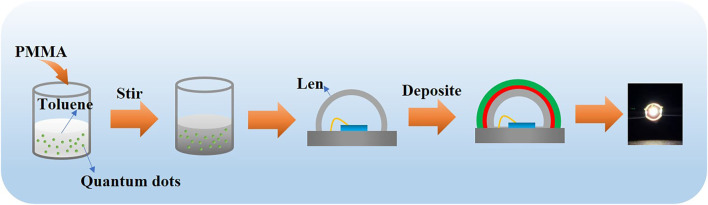
Fabrication process of the CdZnSeS/ZnSeS QD WLED device. QD, quantum dot; WLED, white light-emitting diode.

**FIGURE 5:**
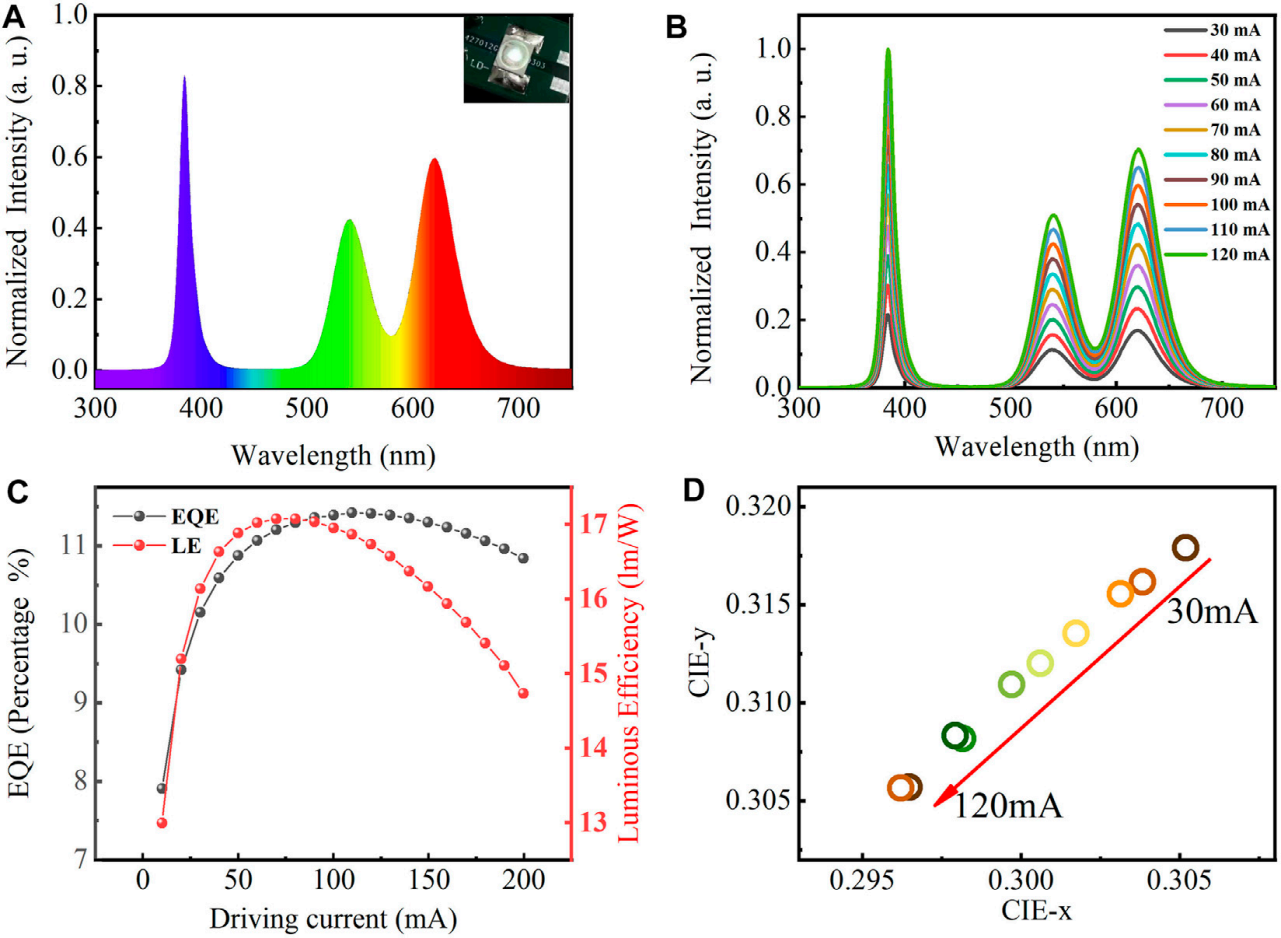
**(A)** Emission spectra of QD LED device driven at 120 mA. **(B)** Emission spectra of QD WLED under different forward currents and driving current-dependent variations in **(C)** EQE and LE and **(D)** CIE. QD, quantum dot; LED, light-emitting diode; WLED, white light-emitting diode; EQE, external quantum efficiency; LE, luminous efficiency; CIE, Commission Internationale de l'éclairage.

The WLED based on the quaternary/ternary alloyed CdZnSeS/ZnSeS QDs is obtained by depositing continuously adjusted quantity ratio of mixture with CdZnSeS/ZnSeS QDs and PMMA. The specific preparation process is shown in Figure 4. The detailed preparation process is mentioned above. Typically, we choose the LED chip with a silicone lens for its high light transmission and uniform light emission. The emission spectra of the as-prepared LED used CdZnSeS/ZnSeS QDs as green and red phosphors with 120-mA driving current at room temperature as depicted in Figure 5A. Three different peaks can be clearly observed for violet GaN-based LED (385 nm), CdZnSeS/ZnSeS green light (531 nm), and CdZnSeS/ZnSeS red light (613 nm).

Figure 5B shows the emission spectra of WLED based on the CdZnSeS/ZnSeS QDs at different forward currents. As the device operating current increases, the emission light intensity of the WLED is gradually enhanced without any visible emission peak shift, indicating that the prepared CdZnSeS/ZnSeS QDs WLED device has good stability under different driving currents and the CdZnSeS/ZnSeS QDs are not saturated. Figure 5C shows the performance of the device. As the driving current varies from 10 to 200 mA, the EQE and LE both increase first and then decrease. As a result of the droop effect caused by carrier overflow, among them, EQE reaches a maximum of 14.86% at 110 mA, and LE reaches a maximum of 28.14 lm/W at 70 mA. The LE and EQE of the UV chip used in this work are 0.3 lm/W and 20%, respectively, so the light conversion efficiency of the CdZnSeS/ZnSeS QDs in the device can be obtained as more than 70%.

EQE is defined as the ratio of the number of photons emitted from the active region per second and the number of electrons injected into LED per second:

$$EQE=\frac{\frac{P}{\left(hv\right)}\;}{I/e}$$ (3)

where P is optical power emitted from the free region and ***I*** is the injection current.

LE can be calculated by the following equation (Zhu et al., 2010):

$$LE=\frac{\Phi }{P_{in}}=\frac{\Phi }{V_{in}\cdot I_{in}}$$ (4)

where  Φ  is the luminous flux of LED and $P_{in\;}$ is the input power, which can be calculated by multiplying the input voltage $V_{\mathrm{in}}$ and the corresponding current $I_{\mathrm{in}}$ .

As shown in Figure 5D, the device reaches a corresponding color coordinate (0.305, 0.317) at 30 mA, located in the white light-emitting area. Under different forward bias currents, the corresponding color coordinates of the as-prepared LEDs verify from (0.305, 0.317) to (0.296, 0. 305) with slight changes, which shows that WLEDs have good color stability. The slight shift of the color coordinate corresponding to blue light can be attributed to the thermal quenching of the light emission of the QDs as a result of the temperature of the LED chip emerging with the increase of the forward current.

**FIGURE 6:**
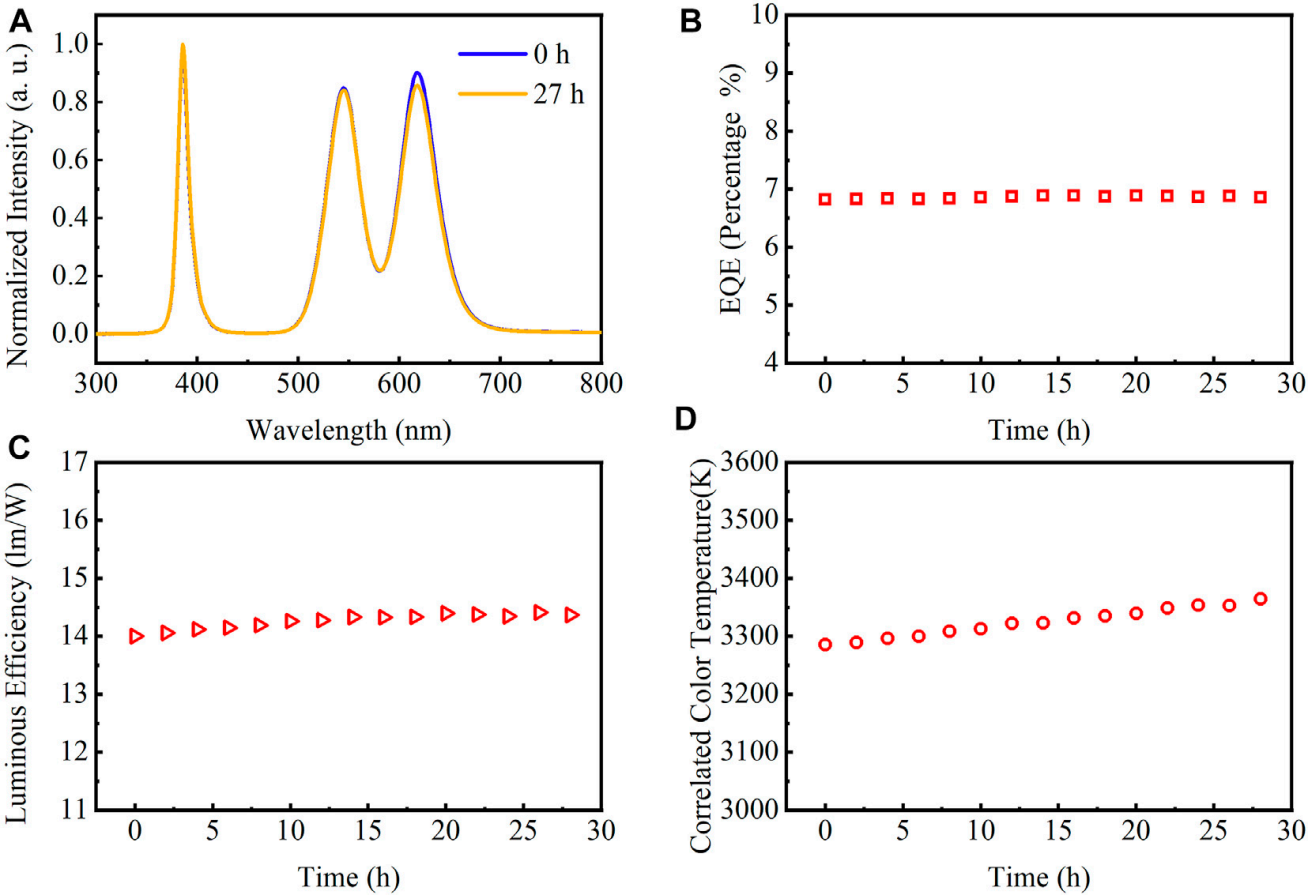
**(A)** Emission spectra of QD WLED at the start of light emission and after 27 h, **(B)** EQE, **(C)** LE, and **(D)** CCT of QD WLED at different time intervals at 3-V voltage. QD, quantum dot; WLED, white light-emitting diode; EQE, external quantum efficiency; LE, luminous efficiency; CCT, correlated color temperature.

To demonstrate the time stability of the device, we perform a time stability experiment of device performance parameters with a working time of 27 h under a forward voltage of 3 V. Figure 6A shows the spectra of the as-papered device before and after 27 h working in that only the red light drops slightly after 27 h, indicating that it has good stability. Since it takes a certain time for the device to reach a stable state, the performance parameters will rise slightly as time increases. Long time operation of the QD WLED at a stable voltage of 3 V reveals that the EQE increased from a maximum of 6.81% to 6.85%, which only increased by 0.5% (Figure 6B). The LE of the device increased from a maximum of 13.99 to 14.36 lm/W, which only increased by 0.37 lm/W (Figure 6C). The CCT increased from 3,285 to 3,364 after 27 h, i.e., only increased 2.4% to its initial value (Figure 6D). These indicate that the thick shell layer CdZnSeS/ZnSeS QDs can maintain good stability during the long-term operation of the device and keep the LE and CCT at an ideal level.

## Conclusion

In summary, the quaternary/ternary alloyed CdZnSeS/ZnSeS QDs were synthesized with emission wavelengths at 531 and 613 nm, with suitable emission line widths (FWHM ∼38 nm). We have demonstrated that the QDs have excellent luminescence performance and thermal stability, fitted well as a luminescent material. The LED integrated with the CdZnSeS/ZnSeS QDs covers the visible spectrum, delivering a LE of 28.14 lm/W, an EQE of 14.86%, and warm bright sunlight close to the spectrum of daylight (CIE coordinates 0.305, 0.317). In particular, as the lighting time increased, the PL intensity, LE, EQE, and CCT of the as-prepared device remained relatively stable with only slight changes. We believe that the device-grade CdZnSeS/ZnSeS QDs with superior optical properties hold great promise for lightings and displays.

## Data Availability

The original contributions presented in the study are included in the article/supplementary material, Further inquiries can be directed to the corresponding authors.
